# Chondroblastoma-Like Soft-Tissue Chondroma of the Hand: A Case Report

**DOI:** 10.7759/cureus.19467

**Published:** 2021-11-11

**Authors:** Ali H AlYami

**Affiliations:** 1 Department of Surgery, Ministry of the National Guard - Health Affairs, Jeddah, SAU; 2 Department of Surgery, King Saud Bin Abdulaziz Universty for Health Sciences, Jeddah, SAU

**Keywords:** palmar mass, excisional tissue biopsy, oncology, soft-tissue chondroma, chondroblastoma-like chondroma

## Abstract

We present a case of chondroblastoma-like chondroma (CLC) of soft tissue that manifested as a palmar mass on the left hand of an 84-year-old man. Physical examination, imaging investigation, and needle biopsy initially suggested a giant cell tumor of the tendon sheath, until a correct diagnosis was made based on excised specimen histology. The patient underwent a marginal excision for soft-tissue mass removal. Follow-up examination showed excellent relief of symptoms, and full hand mobility was regained. The diagnosis of CLC is challenging because the radiographic and pathological features vary. Therefore, we highly emphasize the importance of utilizing excisional tissue for histopathological assessment in atypical chondroma presentations.

## Introduction

Soft-tissue or extraskeletal chondromas are rare, slow-growing, benign neoplasms, which are predominantly formed by the hyaline cartilage [1]. These tumors typically arise from tenosynovial sheaths or soft tissue adjacent to tendons [2]. Hands and feet are the most frequently involved sites [1]. However, less frequent sites, such as the knee, trunk, oral cavity, pharynx, and the base of the skull, have also been reported in the literature [3-5].

Although most soft-tissue chondromas are completely composed of the lobular, mature hyaline cartilage, different histological variants have been recorded [2], including those with chondroblastoma-like features, such as hypercellularity, variable numbers of interspersed osteoclast-like multinucleated giant cells, and an eosinophilic chondroid matrix [2]. It is highly important that these benign tumors be distinguished from more aggressive chondroid neoplasms to spare the patient from unnecessary radical therapy. To the best of our knowledge, only a few case reports of the chondroblastoma-like chondroma (CLC) variant have been published in the literature [6]. In this study, we present the case of an 84-year-old male with CLC that manifested as a palmar mass on his left hand.

## Case presentation

An 84-year-old man with diabetes mellitus, hypothyroidism, ischemic heart disease, and benign prostatic hyperplasia on medical treatment was referred to our clinic with the complaint of a painless mass in the palmar aspect of his left hand. Hand function was affected, and the patient had difficulty in gripping and holding objects for approximately one year. No constitutional symptoms were present, and there was no history of malignancy.

On physical examination, a mass was found in the volar aspect of his left hand (zone 2), over the second and third metacarpals and the metacarpophalangeal joint (MCPJ), with a normal overlying skin. The mass was well defined, firm, fixed, nontender on palpation, and measured 3 × 2.5 cm in diameter. The digital range of motion and neurovascular examination findings were normal.

**Figure 1:**
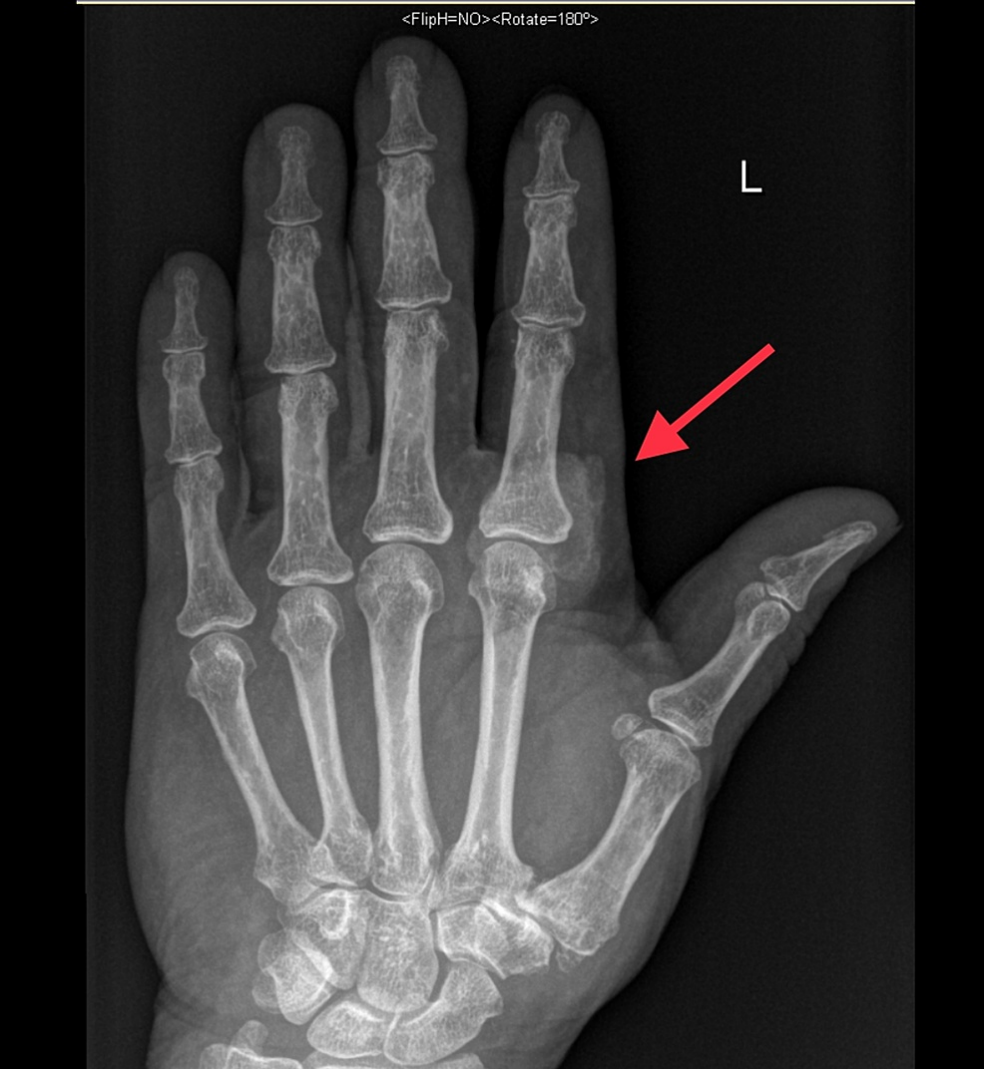
Plain radiograph (anteroposterior view) showing a heterogeneous soft-tissue swelling with calcification in the anterior aspect of the second MCPJ. MCPJ: metacarpophalangeal joint.

**Figure 2:**
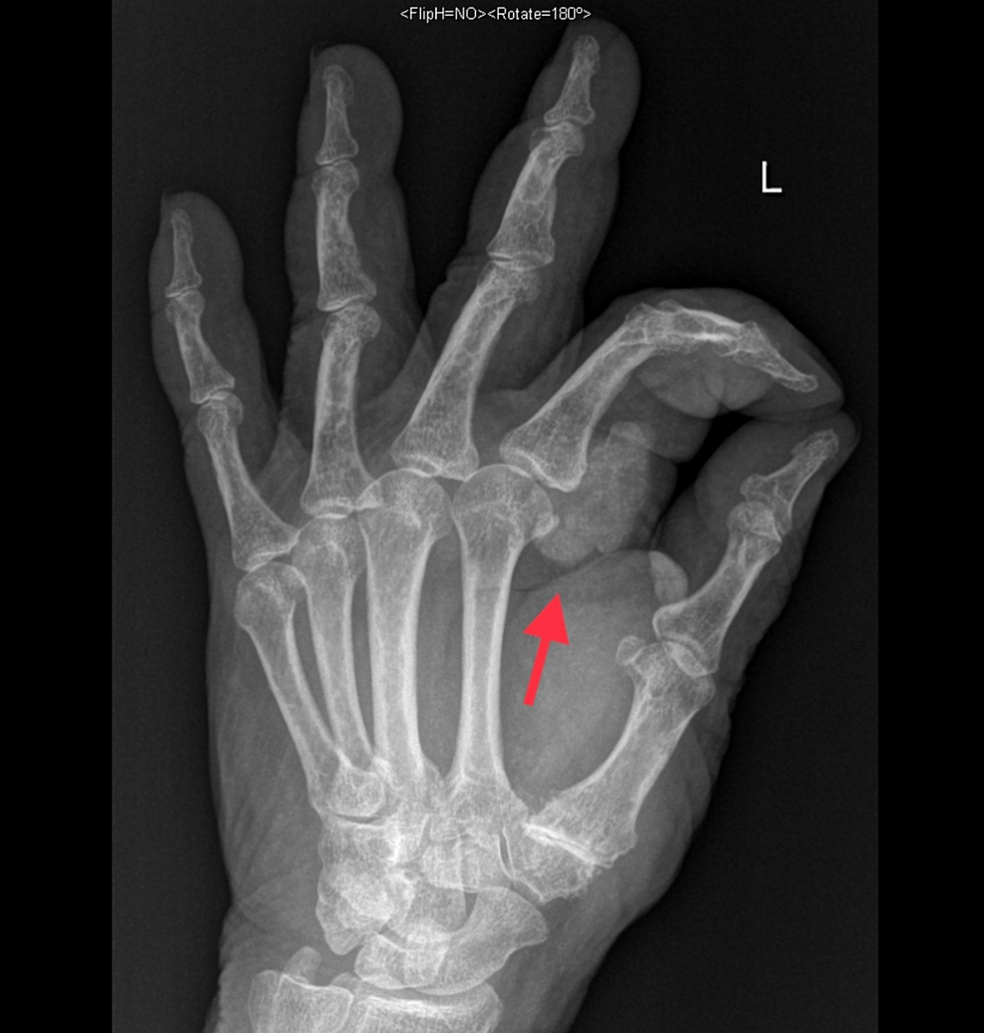
Plain radiograph (oblique view) showing a heterogeneous soft-tissue swelling with calcification in the anterior aspect of the second MCPJ. MCPJ: metacarpophalangeal joint.

**Figure 3:**
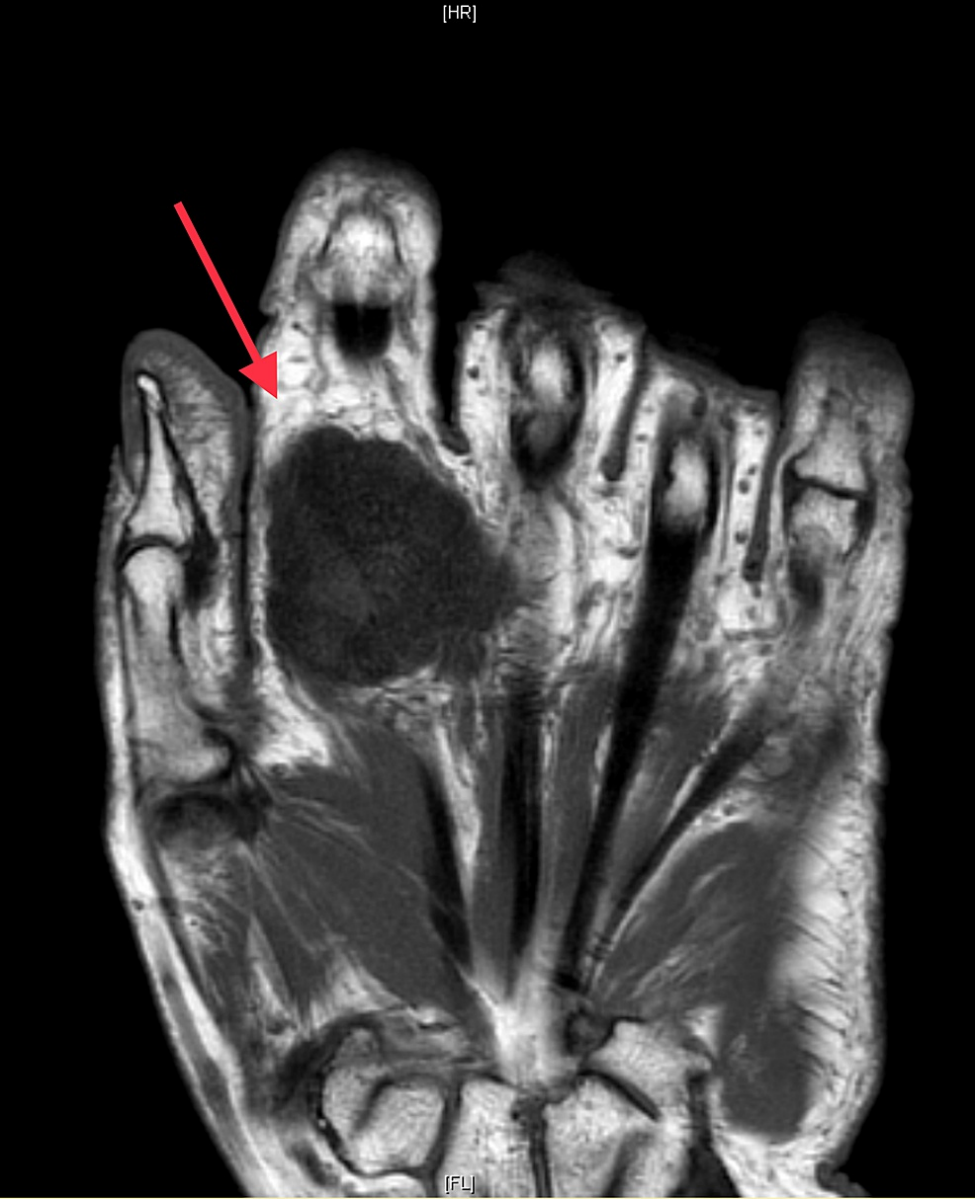
T1-weighted MR images demonstrating an intermediate signal, with a few areas of a low signal, and a large, well-defined, lobulated, oval-shaped mass measuring 3.0 cm in proximal–distal, 2.6 cm in anteroposterior, and 3.1 cm in medial–lateral dimensions. The mass is present in the deep subcutaneous soft tissues of the palmar aspect of the hand, at the level of the second proximal phalanx, in a close proximity to the second flexor digitorum tendon. MR: magnetic resonance.

**Figure 4:**
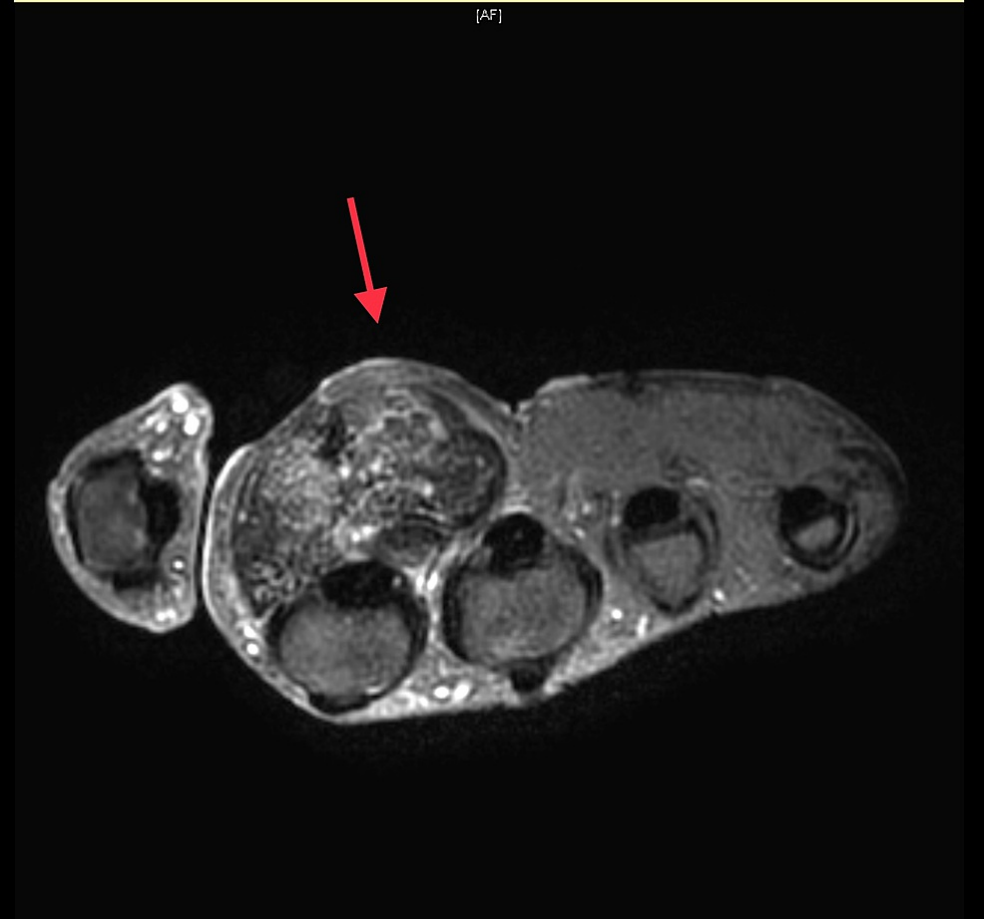
T2-weighted MR images showing heterogeneous, mixed low and high signal intensity. MR: magnetic resonance.

**Figure 5:**
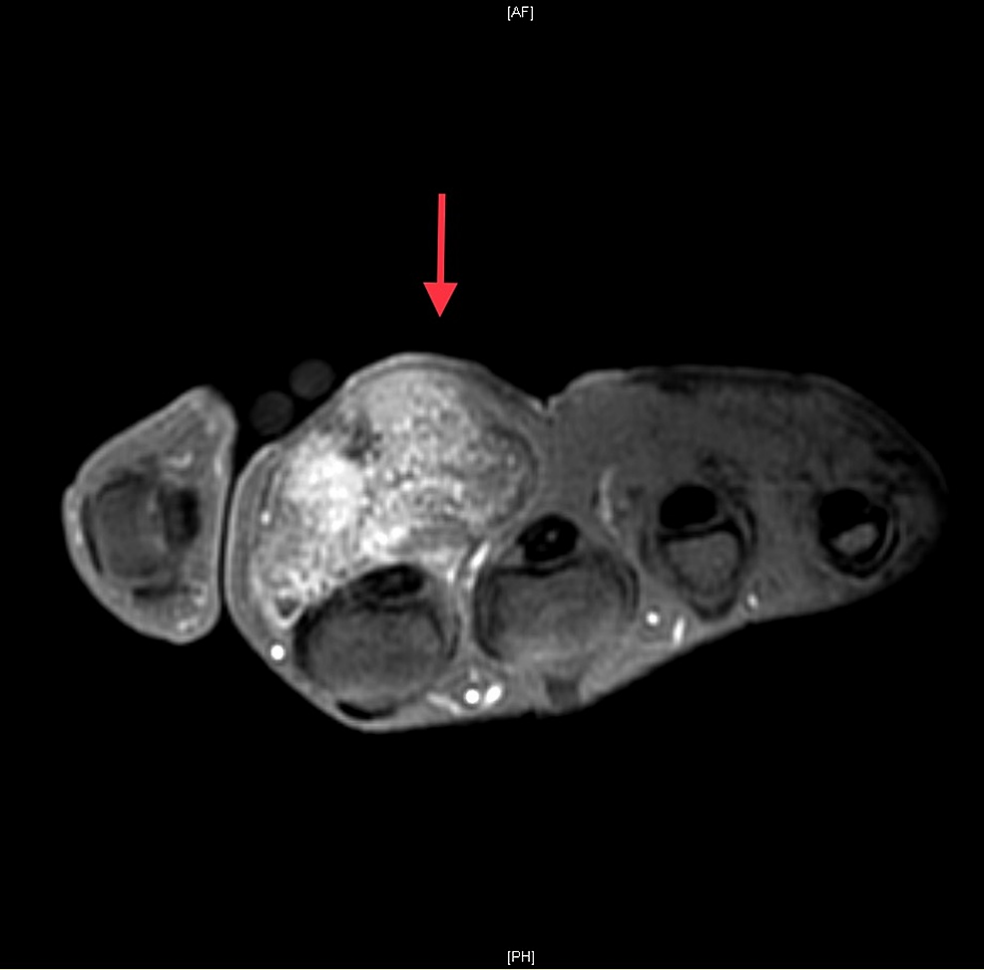
MR image following IV gadolinium administration, with obvious heterogeneous enhancement. IV: intravenous; MR: magnetic resonance.

Plain radiographs in anteroposterior and oblique views demonstrated a heterogeneous soft-tissue swelling with calcification in the anterior aspect of the second MCPJ (Figures 1, 2). Moreover, MRI demonstrated a large, well-defined, lobulated, oval-shaped mass measuring 3.0 cm in proximal-distal, 2.6 cm in anteroposterior, and 3.1 cm in medial-lateral dimensions. The mass was present in the deep subcutaneous soft tissues of the palmar aspect of the hand, at the level of the second proximal phalanx, in close proximity to the second flexor digitorum tendon (Figure 3). Nevertheless, the flexor digitorum profundus and flexor digitorum superficialis tendons were intact, and no perilesional edema or invasion was observed. The second metacarpal head and proximal second phalanx showed a normal bone marrow signal. T1-weighted images demonstrated an intermediate signal, with a few areas of a low signal (Figure 3). T2-weighted images showed heterogeneous, mixed low and high signal intensity. Following intravenous gadolinium administration, heterogeneous enhancement was obvious (Figures 4, 5).

A core needle biopsy showed the proliferation of predominantly round cells, with a low to moderately abundant eosinophilic cytoplasm. The nuclei were ovoid and showed nuclear grooves, and the matrix was predominantly chondroid with foci of coarse granular calcification. These features were suggestive of a chondroid process and justified a surgical resection of the entire lesion for proper classification and characterization.

**Figure 6:**
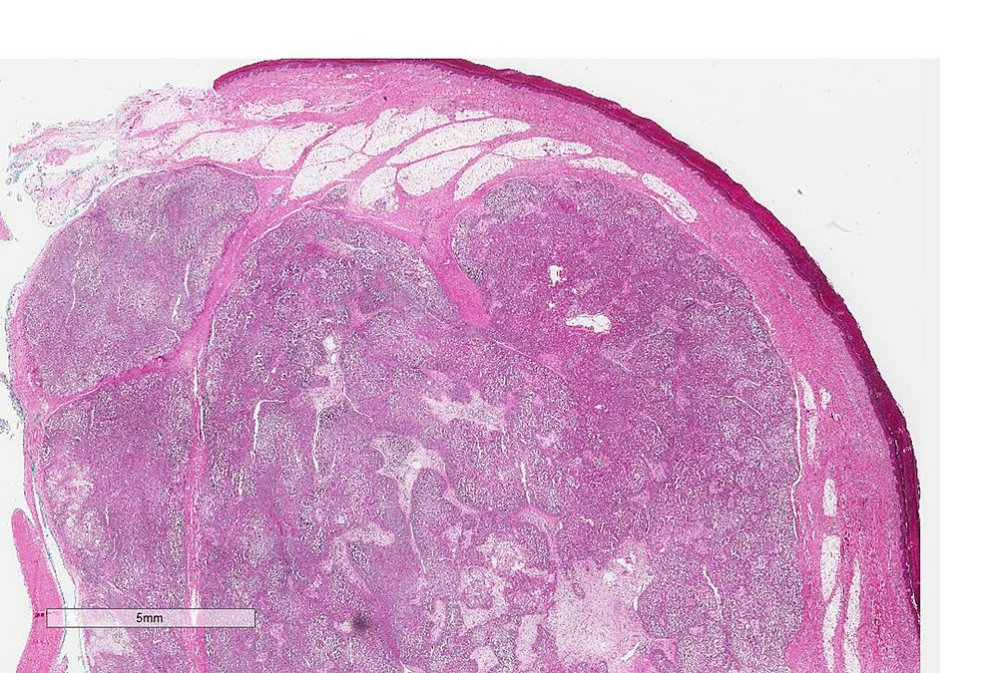
Well-circumscribed tumor with a lobular growth pattern.

**Figure 7:**
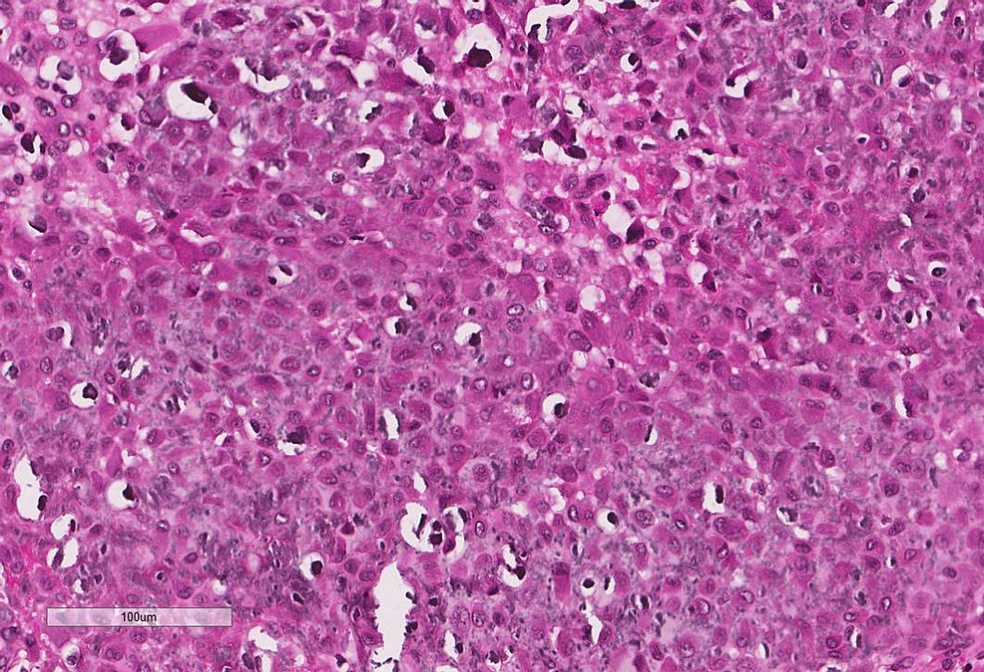
Cellular areas showing large polygonal cells with grooved nuclei and a moderately abundant eosinophilic cytoplasm.

**Figure 8:**
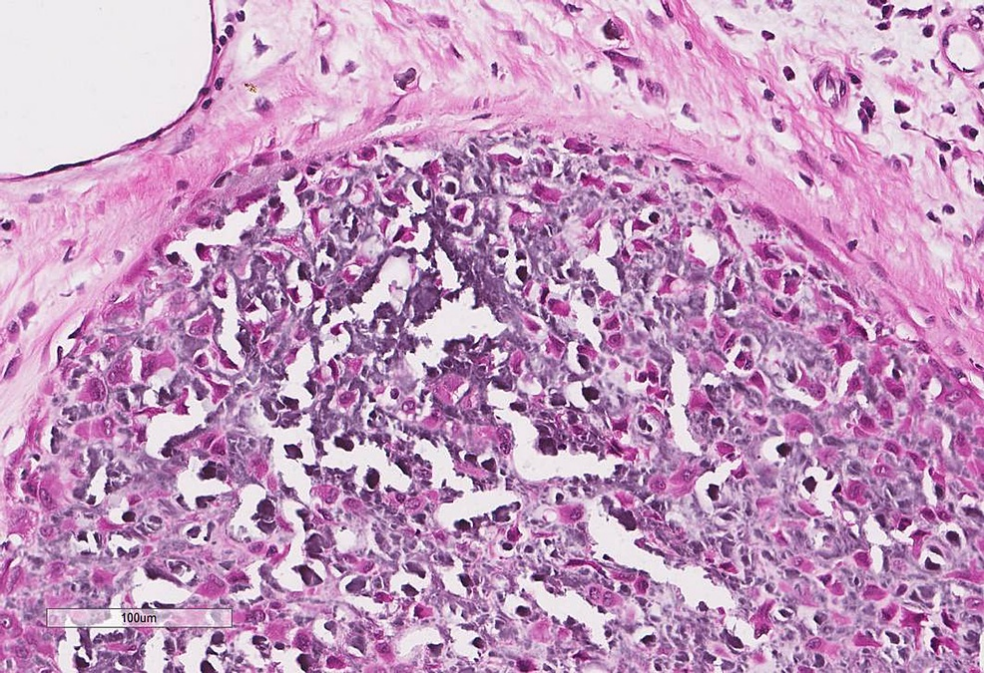
Foci of chicken-wire calcification surrounding individual lacunae.

The patient was admitted and underwent a marginal excision of the soft-tissue mass, based on the results of the core needle biopsy, through a palmar incision over the apex of the mass and an extended Z-shaped incision at the distal palmar crease. Resection of the tumor revealed a yellow-tan, well-circumscribed solid lesion measuring 4 × 4 × 2 cm, which was carefully dissected and completely removed as a single mass. The digital nerve of the index finger and vessels was identified and dissected away from the mass. It was attached to the paratenon of the second flexor tendon, which was saved and protected. A careful homeostasis was achieved. The closure was performed in layers, and a soft dry dressing was applied. The surgery was uneventful, with no surgical or postoperative complications. The final report of the histological sections showed a well-circumscribed and markedly lobulated cellular neoplasm, composed of chondroblast-like cells with round to oval nuclei and a moderately abundant eosinophilic cytoplasm. The nuclei had notches and small but prominent nucleoli. A few osteoclast-like giant cells, as well as prominent chicken-wire calcifications, were noted. There was no frank cytological atypia, necrosis, or increased mitotic activity (Figures 6, 7, 8). As all these findings were consistent with the diagnosis of CLC, the patient diagnosis was modified to CLC of the hand. In the postoperative period, the patient was encouraged to use his hand as tolerated and was discharged home in good condition.

The patient was routinely followed up in the clinic after surgery and started hand-strengthening and range-of-motion exercises with a physiotherapist. Follow-up examination of the patient showed excellent relief of symptoms. He regained full mobility of the hand; the wound was healed, and the stitches were removed. Upon follow-up, there was no clinical or radiographic evidence of tumor recurrence, and the patient remained free of symptoms.

## Discussion

Chondromas of soft tissue are rare, non-malignant tumors that constitute 1.5% of total soft tumors [6]. Most of these neoplasms grow in the extremities, with more than 50% occurring in phalanges [6]. Unfortunately, because of the lack of data, the prevalence of this exact CLC variant has not yet been estimated. However, according to Cates et al. [2], eight out of 17 (47%) soft-tissue chondroma cases were reported to be CLCs. Moreover, the mean age of the patients at diagnosis was 46 (range: 28-71) years, with an average mass size of 2.3 (range: 1.5-2.8) cm [2]. In our case, the patient was older and had a larger mass at presentation.

CLCs have been macroscopically described as ovoid nodules or multinodular fragments with a tan-pink to tan-yellow color and a firm to rubbery consistency, which is similar to our findings [2,6]. Histologically, CLCs have been observed to grow in a lobular pattern, with a well-circumscribed margin. The chondroblastoma-like areas are not consistent among patients in terms of the magnitude, ranging from 10% to 100% [2]. These chondroblastic foci are composed of chondrocytes of various sizes and are polygonal to elongated in shape [2]. In addition, some are mixed with varying numbers of osteoclast-like multinucleated giant cells [2]. In contrast to mature chondrocytes, in which the nuclei are small and round, with a minimal eosinophilic cytoplasm, chondroblastic cells have nuclei that are eccentrically located and are larger and oval or reniform in shape, with a moderate to abundant eosinophilic cytoplasm [2]. Calcification of the chondroid matrix in a lace-like or chicken-wire pattern is also a commonly reported feature [1,2,5,6]. Cytological atypia and mitotic figures are uncommon and not classically observed, although two cases have been reported with these features [2].

It is unusual for a soft-tissue chondroma in the hand to show calcifications on plain radiographs. Moreover, typical MRI findings for chondromas demonstrate minimal perilesional inflammation and a high T2 signal. As these radiological features were not consistent with our case findings, we proceeded with a core needle biopsy, followed by a marginal excision, to determine the correct pathology.

Based on the literature and our own experience, the recommended management for rare CLCs of the hand is a conservative excision since the clinical behavior of CLC is benign and nonaggressive. However, local hand recurrence has been reported in a few cases during an observation period of 1-10 years following excision [1]. Therefore, we also recommend a routine follow-up plan and assessment to prevent recurrence. After a follow-up of approximately 1 year, our patient remained disease-free without recurrence.

This case illustrates how this condition may represent a diagnostic challenge due to variable radiographic and pathological features. We attempted to highlight the clinical examination, possible diagnostic imaging, and surgical findings, in addition to the more commonly highlighted histopathology. MRI may help differentiate CLC from more common hand tumors, but an excisional tissue diagnosis is needed for clarification. Diagnosis based on core biopsy alone should be avoided because it will likely fail to show the varied microarchitecture that is indicative of CLC.

## Conclusions

CLC is a rare variant of soft-tissue chondroma, which mostly occurs in the hands. Owing to its expression as secondary degenerative features, the diagnosis of this condition is challenging. Therefore, we highly emphasize the importance of utilizing excisional tissue for histopathological assessment in atypical chondroma presentations.
